# Seasonal and inter-annual drivers of yellow fever transmission in South America

**DOI:** 10.1371/journal.pntd.0008974

**Published:** 2021-01-11

**Authors:** Arran Hamlet, Katy A. M. Gaythorpe, Tini Garske, Neil M. Ferguson

**Affiliations:** MRC Centre for Global Infectious Disease Analysis, Department of Infectious Disease Epidemiology, Imperial College London, London, United Kingdom; Johns Hopkins Bloomberg School of Public Health, UNITED STATES

## Abstract

In the last 20 years yellow fever (YF) has seen dramatic changes to its incidence and geographic extent, with the largest outbreaks in South America since 1940 occurring in the previously unaffected South-East Atlantic coast of Brazil in 2016–2019. While habitat fragmentation and land-cover have previously been implicated in zoonotic disease, their role in YF has not yet been examined. We examined the extent to which vegetation, land-cover, climate and host population predicted the numbers of months a location reported YF per year and by each month over the time-period. Two sets of models were assessed, one looking at interannual differences over the study period (2003–2016), and a seasonal model looking at intra-annual differences by month, averaging over the years of the study period. Each was fit using hierarchical negative-binomial regression in an exhaustive model fitting process. Within each set, the best performing models, as measured by the Akaike Information Criterion (AIC), were combined to create ensemble models to describe interannual and seasonal variation in YF. The models reproduced the spatiotemporal heterogeneities in YF transmission with coefficient of determination (R^2^) values of 0.43 (95% CI 0.41–0.45) for the interannual model and 0.66 (95% CI 0.64–0.67) for the seasonal model. For the interannual model, EVI, land-cover and vegetation heterogeneity were the primary contributors to the variance explained by the model, and for the seasonal model, EVI, day temperature and rainfall amplitude. Our models explain much of the spatiotemporal variation in YF in South America, both seasonally and across the period 2003–2016. Vegetation type (EVI), heterogeneity in vegetation (perhaps a proxy for habitat fragmentation) and land cover explain much of the trends in YF transmission seen. These findings may help understand the recent expansions of the YF endemic zone, as well as to the highly seasonal nature of YF.

## Author summary

Yellow fever (YF) is a viral haemorrhagic fever found in tropical South America and Africa that affects both humans and non-human primates (NHPs). Despite a long-standing recognition of YF as a pathogen of significant public health concern, not much is known about why cases are reported in some years and not others, or what drives the strong seasonal trends that are observed in South America.

Using a combination of statistical techniques, the authors have looked at the relationship between different types of land-use, vegetation, climate, human and NHP and the reporting of YF across all of South America and both inter-annual and seasonally over the period 2003–2016. The authors have found that both are highly influenced by changes in vegetation, however the inter-annual is additional influenced by land-cover and the seasonal climatic factors.

The authors have found that the differing drivers of seasonal and inter-annual transmission, though both are predominantly shaped by changes in vegetation, highlighting the role of land-cover in influencing the inter-annual and climate the seasonal.

This research enhances our understanding of YF transmission in South America, describing the geographic and temporal distribution of cases in relation to vegetation, land-cover and climate. This research is of particular importance, given the recent large-scale outbreaks of YF across the continent, global YF vaccination shortages and the exportation of cases globally.

## Introduction

Disease transmission is influenced by both intra- and interannual variations in weather and the environment, particularly for vector-borne pathogens [1–3]. These deviations may be relatively short in duration, due to seasonal changes in weather [2] or phenomena such as El Niño [4], or they may represent a more persistent change, due to climate change [5] or alterations to land-cover [6]. While climate change is likely to alter both the distribution and intensity of a number of diseases [7–9], this process takes place over a substantially longer period of time than anthropogenic land conversion which can completely change large swathes of natural habitat in a few years [10,11]. Rapid habitat change is often associated with disease occurrence [12], especially of zoonotic infections [13], potentially due to an increased interaction between sylvatic reservoirs and humans, expressly at intermediate levels of transformation [14].

Yellow fever (YF) is a zoonotic disease caused by the yellow fever virus (YFV), a flaviviridae arbovirus infecting both humans and non-human primates (NHPs) [15]. Originating in Africa, YF spread to South America with the slave trade [16] and is currently endemic in 34 countries in Africa and 13 in South America [17]. In South America, YFV transmission occurs in two cycles, the sylvatic and urban. In the former, transmission is maintained by sylvatic mosquito species of the *Haemogogus* and *Sabethes* genera between NHPs, with humans considered incidental hosts. If the virus establishes itself in the domestic *Aedes aegypti*, also a vector of both dengue and Zika viruses, transmission can be sustained in the absence of a NHP reservoir. This can cause large and explosive outbreaks, the latest being the 2015–2016 outbreak in Angola and the Democratic Republic of the Congo, the largest in the past 30 years [18].

In South America the sylvatic cycle has accounted for almost all cases since 1942 [19], and has historically been confined to Amazonian regions. However, over the past 20 years the area where YF is endemic in NHPs has seen rapid geographic expansion. In Brazil this has resulted in 5 reassessments of the zone of YF endemicity since 2000, with the latest update in 2018 including the entire country [20,21]. The reasons for this expansion are unknown. Furthermore, outbreaks in Brazil’s South-East Atlantic forest in 2016–2017 and 2017–2018 have been the largest ever recorded in the country, in humans and NHPs, with cases reported in states that have never previously recorded YF [22]. While there is no evidence of urban transmission in these outbreaks, confirmed human and NHP cases in the vicinity of Brazil’s largest cities–areas with high *Aedes aegypti* density–is a cause for concern [23,24]. In addition to a lack of understanding of these interannual drivers of transmission, there is a general dearth of understanding of the seasonality of YF. Despite the seasonality of YF having long been well established [25,26], there has been little research on quantifying the associated environmental and climatic drivers of seasonality in South America

We investigated the drivers of YF transmission both intra and interannually, using temporally varying covariates related to climate, land-cover, vegetation and human and NHP demographics. Covariates related to these were selected based on their previously demonstrated roles in vector-biology, increased suitability for disease transmission or relationships with YF [27–30]. In particular, we examined the role of vegetation cover and its heterogeneity/fragmentation. While the influence of habitat fragmentation in YFV transmission has previously been postulated [14,31,32], detailed research into the role habitat fragmentation and land-cover play in YFV sylvatic spillover is absent.

By utilising two model structures, we investigated the differential drivers of seasonal, and interannual variation in YF incidence. These models were fit to the number of months reporting YF at each administrative level 1 geographic unit (for example, the province or state), using exhaustive model fitting in hierarchical negative-binomial regressions. The best performing models, as defined by the Akaike Information Criterion (AIC) were weighted and combined using Akaike weights to produce an ensemble model [33]. Model robustness was confirmed using spatial block bootstrapping.

## Materials and methods

### YF data

Reports of YF cases in humans were assembled from various sources, including the Weekly Epidemiological Record [34], Disease Outbreak News [35], and the Pan American Health Organization [36] for the period 2003–2016. Only reports where the month of symptom onset was recorded were included (823 of the original 1073 reports), and these were geo-located to the first sub-national administrative level, here termed province.

Two datasets were derived from our report database. For each, we classified each month (over the 14 years of the data) for each province as a report month if one or more YF cases had onset dates in that month. This resulted in 397 report months over 165 unique provinces. For the interannual analysis, numbers of report months were summed within each year for each province–giving a dataset of the number of report months for each province for each of the 14 years considered. For the seasonal dataset, numbers of report months were summed over years for each province and each of the 12 months of the calendar year.

The inter-annual dataset, which uses the calendar year is a simplification of long term (multi-year) transmission patterns. Disease transmission likely does not confirm fully to these demarcations of years, and so may not be fully captured by our usage of the calendar year for inter-annual transmission. However, in the absence of previously defined seasonal patterns for each administrative location (which will likely change across the region of study) we have defaulted to the current World Health Organization/Pan American Health Organization format which uses the simple calendar year [36].

### Covariates

In total, 19 covariates were considered (Table 1). These were selected based on knowledge of the biology and distributions of vector species, host dynamics, inferences from the role of land-cover change and vegetation heterogeneity and the epidemiology of yellow fever in South America [2,30,37,38]. For the temporally changing covariates in the seasonal model, the values of the first year were subtracted from the final year of study (2003 and 2016 respectively), for the interannual covariates, we subtracted the value of the one year from the next year (i.e. 2003 land cover– 2002 land cover produced the 2003 land cover temporal change).

**Table 1:** Table of classification types and the covariates included.

Covariate	Classification	Temporal resolution	Contributing covariate types	Description	Reference
Monkey species (count)	Host population	Static		The number of monkey species	(41)
Logarithm of human population	Host population	Static		The logarithm of human population	(39)
Change in logarithm of human population		Static		The change in the logarithm of human population from 2003 to 2016	
Enhanced Vegetation Index (EVI) (0–1)	Vegetation	Annual/monthly		A vegetation index designed to improve sensitivity in high biomass regions	(43)
EVI amplitude	Vegetation	Annual/monthly		The amplitude of the EVI	
Day temperature (°C)	Climate	Annual/monthly		The day temperature	(42)
Day temperature amplitude (°C)	Climate	Annual/monthly		The amplitude of the day temperature	
Rainfall	Climate	Annual/monthly		The rainfall	(44)
Rainfall amplitude	Climate	Annual/monthly		The amplitude of the rainfall	
Forest cover	Land-cover	Annual	MCD12Q1 –Value 1	The proportion of the administrative unit covered by Evergreen Needleleaf forest	(43)
		Annual	MCD12Q1 –Value 2	The proportion of the administrative unit covered by Evergreen Broadleaf forest	
		Annual	MCD12Q1 –Value 3	The proportion of the administrative unit covered by Deciduous Needleleaf forest	
		Annual	MCD12Q1 –Value 4	The proportion of the administrative unit covered by Deciduous Broadleaf forest	
		Annual	MCD12Q1 –Value 5	The proportion of the administrative unit covered by Mixed forest	
Savanna cover	Land-cover	Annual	MCD12Q1 –Value 8	The proportion of the administrative unit covered by Woody savanna	
		Annual	MCD12Q1 –Value 9	The proportion of the administrative unit covered by Savanna	
Cropland/natural vegetation mosaic cover	Land-cover	Annual	MCD12Q1 –Value 14	The proportion of the administrative unit covered by cropland/natural vegetation mosaic	
Urban cover	Land-cover	Annual	MCD12Q1 –Value 13	The proportion of the administrative unit covered by urban areas	
Cropland cover	Land-cover	Annual	MCD12Q1 –Value 12	The proportion of the administrative unit covered by cropland	
Forest cover temporal change	Land-cover change	Annual		Interannual model: The current year landcover–previous year landcover value Seasonal model: The final year (2016)–the first year (2003) landcover value	
Savanna cover temporal change	Land-cover temporal change	Annual			
Natural vegetation/cropland mosaic cover temporal change	Land-cover temporal change	Annual			
Urban cover temporal change	Land-cover temporal change	Annual			
Cropland cover temporal change	Land-cover temporal change	Annual			
Vegetation heterogeneity	Vegetation heterogeneity	Annual/monthly		This is the standard deviation of the EVI at a 1x1km resolution within the administrative unit	
Vegetation heterogeneity temporal change	Vegetation heterogeneity temporal change	Annual/monthly		Interannual model: The current year landcover–previous year vegetation heterogeneity value Seasonal model: The final year (2016)–the first year (2003) vegetation heterogeneity	

Covariates were standardised to facilitate comparison through the following formula,

$$z=\frac{x-\mu }{\sigma },$$

where *z*, is the standardised value, *x*, the pre-standardised value, *μ*, the mean of the pre-standardised values and *σ*, the standard deviation of the values. Standardised coefficient values for the ensemble models of the interannual and seasonal models are found in Table 1.

The variable importance refers to how a measurement score decreases when a feature is not available. Initially the full model with the initial dataset is fit, and the R^2^ value calculated. Then the model is refit to a modified initial dataset, where the variable of interest within the dataset been assigned the mean value of that covariate. This is done to produce “dummy data” which creates a covariate that does not provide any useful information. The R^2^ is then calculated from this refit, and the variable importance for variable, *i*, calculated through,

$$variable\;importance_{i}=1-\frac{refit\;R2_{i}}{original\;R2_{i}}.$$

This provides a measure of the feature importance with respect to the original R^2^.

### Host population data

Country and year specific human population sizes were obtained from the UN World Population Prospects [39] and averaged over the study period to obtain average population sizes. Province level estimates of population were obtained by disaggregating this data by using LandScan 2015 [40] population estimates with a 1/120 degree resolution to calculate the proportion of the national population within each province. The mean logarithm of human population over the time-period was used in all seasonal and interannual models. In addition, the relative change in the human population over the 14-year time period was also tested as a covariate (defined as logarithm(population in 2016/population in 2003).

Information on NHP species distribution was obtained through distribution maps of mammals in the western hemisphere [41]. These data were available as demarcations of distribution, which was geo-located to the province level. This was used to calculate the number of NHP species present in each province.

### Climate, vegetation and vegetation heterogeneity data

Datasets, 2003–2016, for temperature [42], enhanced vegetation index (EVI) [43] and rainfall [44] were aggregated to the administrative unit 1 level from their original resolutions (of between 1/120 and 1/12 degree) by calculating population-weighted means, based on the population distribution provided by LandScan 2015 [40]. Here the climate and vegetation data is weighted by the population present, provided by LandScan 2015, and aggregated up to the administrative unit level, this weighting is used to provide climate/vegetation data that is representative of human interaction. The amplitude of the annual cycle Fourier component of these variables was also calculated, to account for the impact of seasonal variation on reports.

Spatial heterogeneity in vegetation was assessed by evaluating the standard deviation of the enhanced vegetation index (EVI) at its original 1/120 degree resolution within an administrative unit.

These covariates were averaged over time using different methods for the interannual and seasonal model datasets. For the seasonal dataset, monthly covariates were provided by taking the mean covariate value in a month across all years 2003–2016 to provide the monthly average over this time-period. For the inter-annual dataset, the mean value of the covariate in a year was used.

### Land-cover data

Land-cover was provided by the MODIS dataset [45], which characterises the dominant land-cover type, 1 of 17, at a grid resolution of 0.8333° globally. This information was aggregated to the province level and the proportion of the province area occupied by each land-cover type calculated. Forest, savanna and shrub land types were summed to provide overall forest and savanna cover. For the seasonal model, the mean land-cover proportions for an administrative unit across the study period (2003–2016) were used. For the inter-annual dataset, land-cover was provided for each year. The inter-annual dataset is included with this submission as S1 Data and the seasonal dataset as S2 Data.

### Regression models

Following initial covariate exploration, a list of covariates identified as relevant to YFV transmission were considered, with log of human population and the fractional change in logarithm human populations included in every model. By considering an exhaustive combination of all 19 covariates, we had 524,288 model structures for the interannual and seasonal frameworks, for a total of 1,048,576 models.

These were fit to either the number of months reporting yellow fever each year (interannual model) or the sum across years of the number of yellow fever reports in each calendar month (seasonal model) using hierarchical negative binomial regression models [46]. A negative binomial model was used due to its appropriateness for measuring count data, and it’s suitability for considering the overdispersion of the data.

Conceptually, hierarchical models are similar to running a standard regression where each row in the dataset refers to an administrative location and a time point (month or year depending on the model structure). By utilising a hierarchical structure however, we can allow parameters to vary between administrative location to avoid introducing biases that arise from treating temporally varying covariates within a location as independent [47]. Here we allow the intercept to vary by administrative location to account for this. These models are shown through the following equations [48,49],

$$Y_{i}|E_{i}∼Poisson(E_{i})$$

$$E_{i}=\mu_{i}$$

$$ln(\mu_{i})=\beta_{0}+\beta_{1}X_{i}+\beta_{1}X_{i}+\cdots \beta_{m}X_{mi}+\varepsilon_{i}$$

$$E_{i}∼Gamma(\lambda_{i},K_{i})$$

Where Y_i_ is the report months of YF in a province, and X_i_ the explanatory covariates and *e*_*i*_, represents the random intercept as defined by the province. E_i_, λ_i_ and K_i_ are the distribution parameters where E_i_ has a Gamma distribution with parameter λ_i_ and K_i_ with the negative binomial distribution, the mean and variance are

$$E(Y_{i})=\mu_{i}$$

$$Var(Y_{i})=\mu_{i}+\frac{\mu_{i}^{2}}{K_{i}}.$$

Models were then ranked base on their Akaike Information Criterion (AIC) and those with an AIC within 3 of the best performing model, as defined as the model with the lowest AIC value, were combined using Akaike weights [33]. To do so, the relative differences in AIC are calculated by,

*Δ_i_* = *AIC_i_*−min(*AIC*) and this is used to obtain an estimated relative likelihood of model, *i*, in proportion to the other models, *k = 1 …K*, included through,

$$w_{i}=\frac{exp\{-\frac{1}{2}\Delta_{i}\}}{\sum_{k=1}^{K}exp\{-\frac{1}{2}\Delta_{k}\}}.$$

The product of each of these model specific weights, *w*_*i*_, and their corresponding model specific predicted values, *p*_*i*_, are summed to generate a single set of weighted predictions, *p*_*A*_,

$$p_{A}=\sum_{k=1}^{K}{w_{k}p}_{k}.$$

Out-of-sample performance was ascertained using a stringent method of cross-validation called spatial block bootstrapping (See S1 Text and S3 Fig).

## Results

### Geographical, seasonal and interannual heterogeneities in YF reports

**Fig 1:**
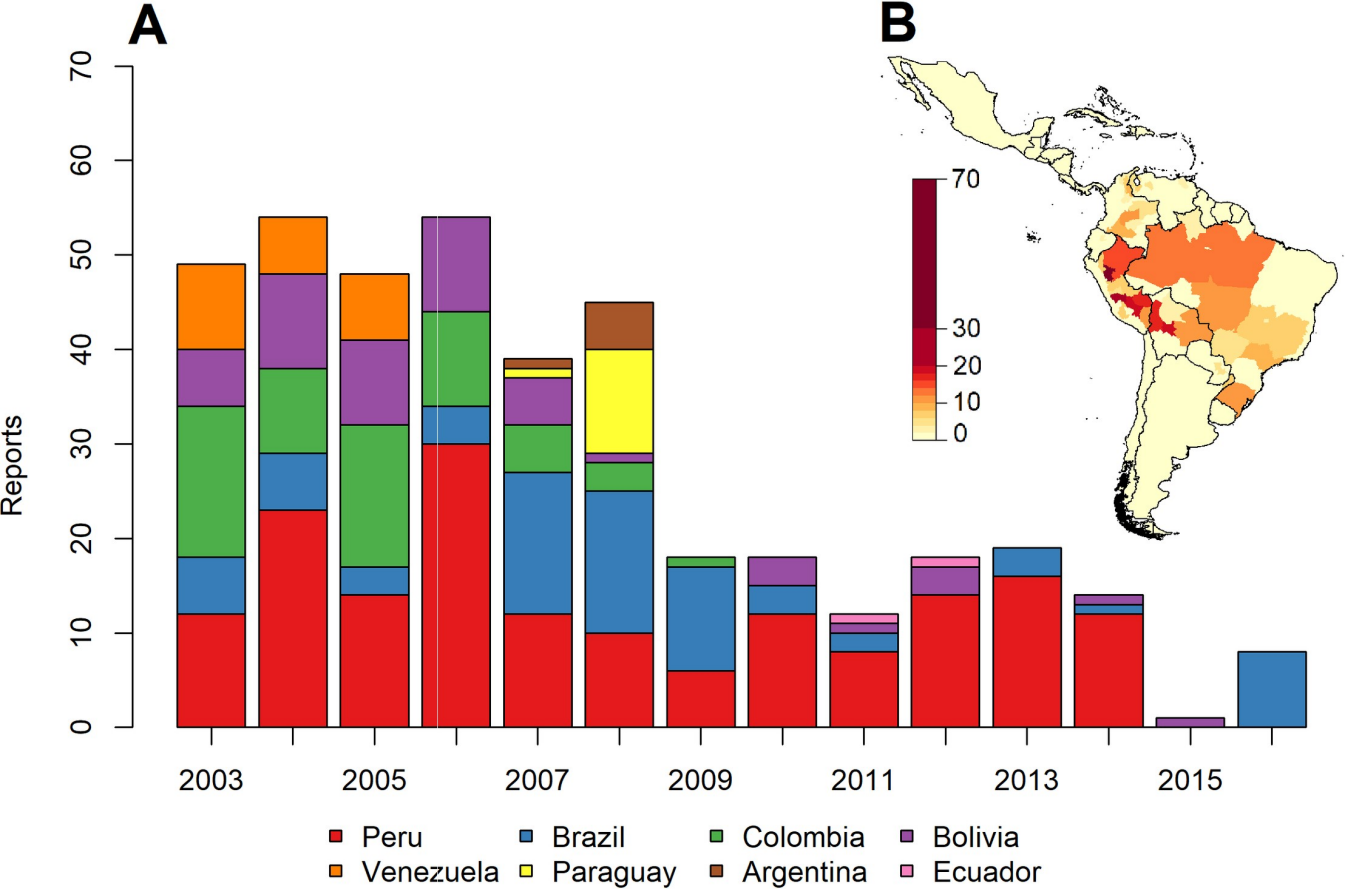
(A) Number of yellow fever report months over time (2003–2016) by country. (B) Total number of yellow fever reports by province (2003–2016) across South America. Figs were produced using the programming language R version 3.5.1 and used publicly available data gathered from the Weekly Epidemiological Record published by the WHO [34].

**Fig 2:**
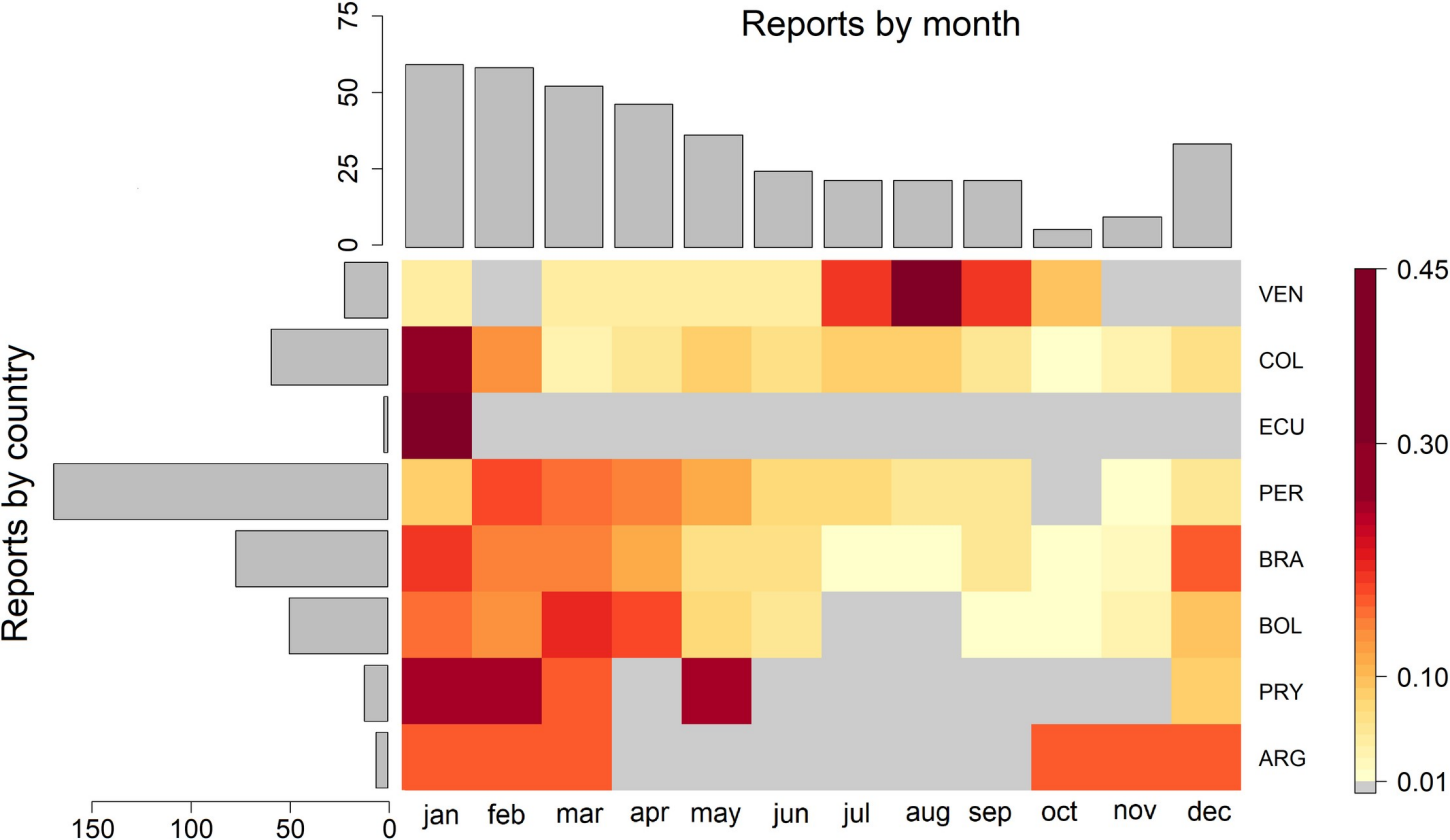
Yellow fever reports by country and month. The heatmap shows the proportion of reports in a country by calendar month, the bar chart on the left-hand side shows the total number of reports by country and the bar chart above shows the total number of months reporting cases by month. Countries are ordered by latitude.

We identified 397 unique months with a report of YF, hereby termed report months (defined spatiotemporally by the administrative unit and month), for the period 2003–2016, in 432 level 1 administrative units across 8 countries (Fig 1). Peru, Colombia and Brazil accounted for 79% of all report months, with Peru alone accounting for 39% (Figs 1A and 2). Within countries, report months show substantial spatial heterogeneity, with a notable clustering in Amazonian regions of Brazil, eastern Peru and Northern Bolivia. States in the South-East Atlantic coast of Brazil have also recorded large numbers of report months (Fig 1B) (21).

The frequency of report months was relatively stable and high during 2003–2008, after which numbers fell, then plateaued until 2015, when they dipped to the lowest level seen with only 1 reported event (Fig 1A). It should be emphasised that report months are a presence/absence indicator and not a proxy for infection incidence. Throughout the endemic zone, YF follows highly seasonal patterns. At the continent scale, transmission is highest from December to February, before dropping to a relatively low level over June to September, and a period of minimal occurrence in October and November (Fig 2). However, this pattern varies slightly by country and latitude (see S2 Text).

### Geographic distributions of model predictions

In total 46 inter-annual and 246 seasonal models had AIC values within 3 of the best (lowest AIC) performing model and so were included in ensemble models.

The ensemble interannual and seasonal models accurately approximate spatiotemporal heterogeneities in YF reports, with coefficient of determination (R^2^) values of 0.43 (95% CI 0.41–0.45) for the interannual and 0.66 (95% CI 0.64–0.67) for the seasonal ensemble predictions. Due to the additional rigour of using spatial block bootstrapping compared with using an entirely random validation set (See SI), out-of-sample prediction R^2^ values were lower at 0.31 (95% CI 0.28–0.34) for the interannual model and 0.45 (95% CI 0.44–0.48) for the seasonal model.

**Fig 3:**
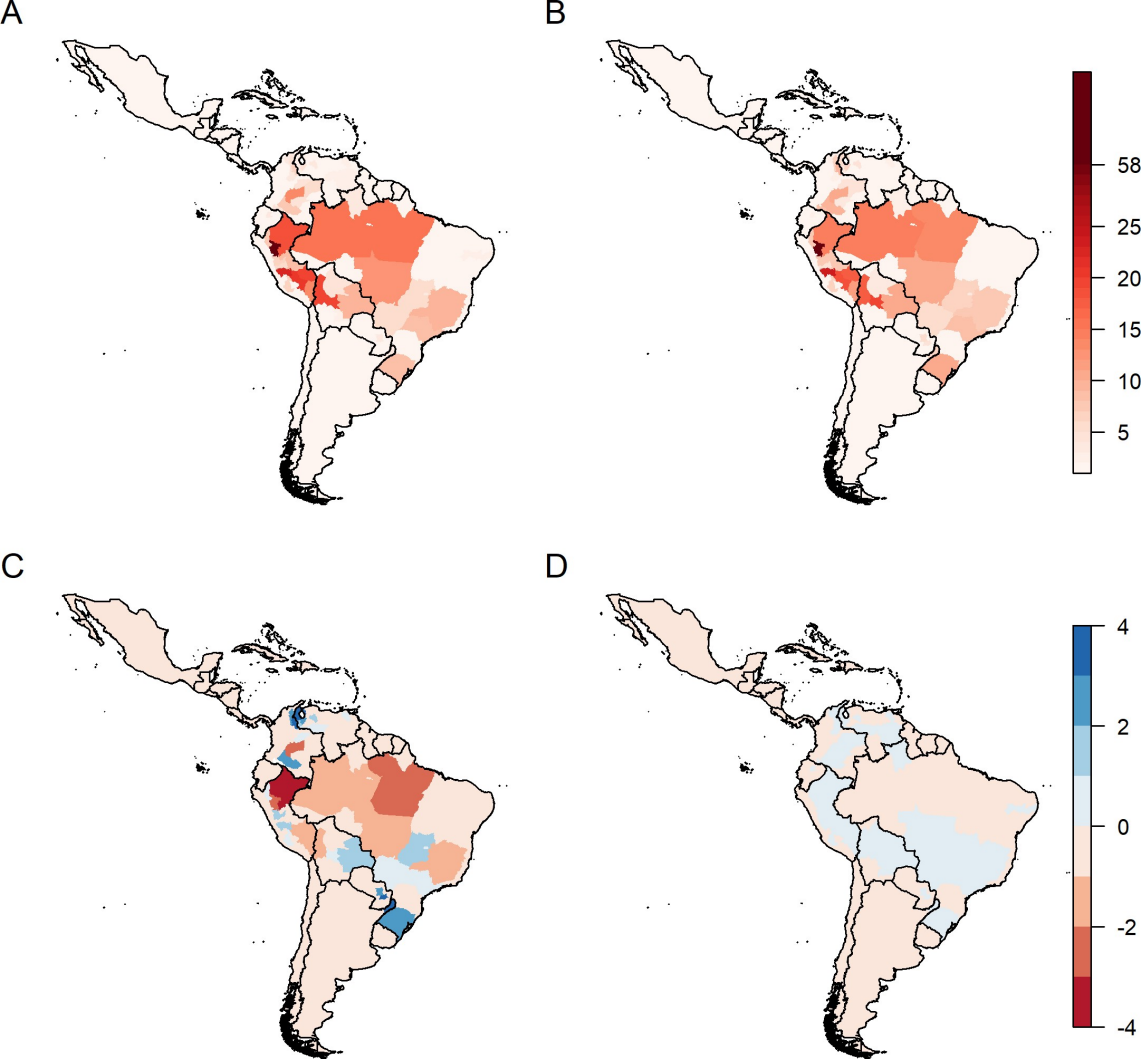
Ensemble model predictions of the number of YF report months for the (A) interannual model and the (B) seasonal model. (C) and (D) show the differences between these predictions and the data for the interannual model and the seasonal model, respectively. Figs were produced using the programming language R version 3.5.1 and the data was generated by the authors.

Model predictions were summed over time for each model to facilitate visual comparison with the data (Fig 3A and 3B vs Fig 1B). Both models reproduce the observed geographic distributions of reports well, though the aggregate ensemble seasonal model predictions give a better fit to the data (Fig 3). Differences between the ensemble interannual model predictions and the data range from -4.35 to +3.08. The model over-predicts reports for much of Eastern Peru and the North-West of Brazil, and predicts fewer reports than observed for Rio Grande do Sul in Brazil, and Misiones province in Argentina. There is additionally a cluster of lower than observed predictions on the Colombian/Venezuelan border. Ensemble seasonal model predictions showed deviations from the data an order of magnitude smaller than seen for the interannual model. The seasonal model slightly underpredicts YF reports, with only Brazilian states in the Amazon, Rio de Janeiro, and the East/North-East of the country predicted as having more reports than observed.

### Temporal distributions of model predictions

**Fig 4:**
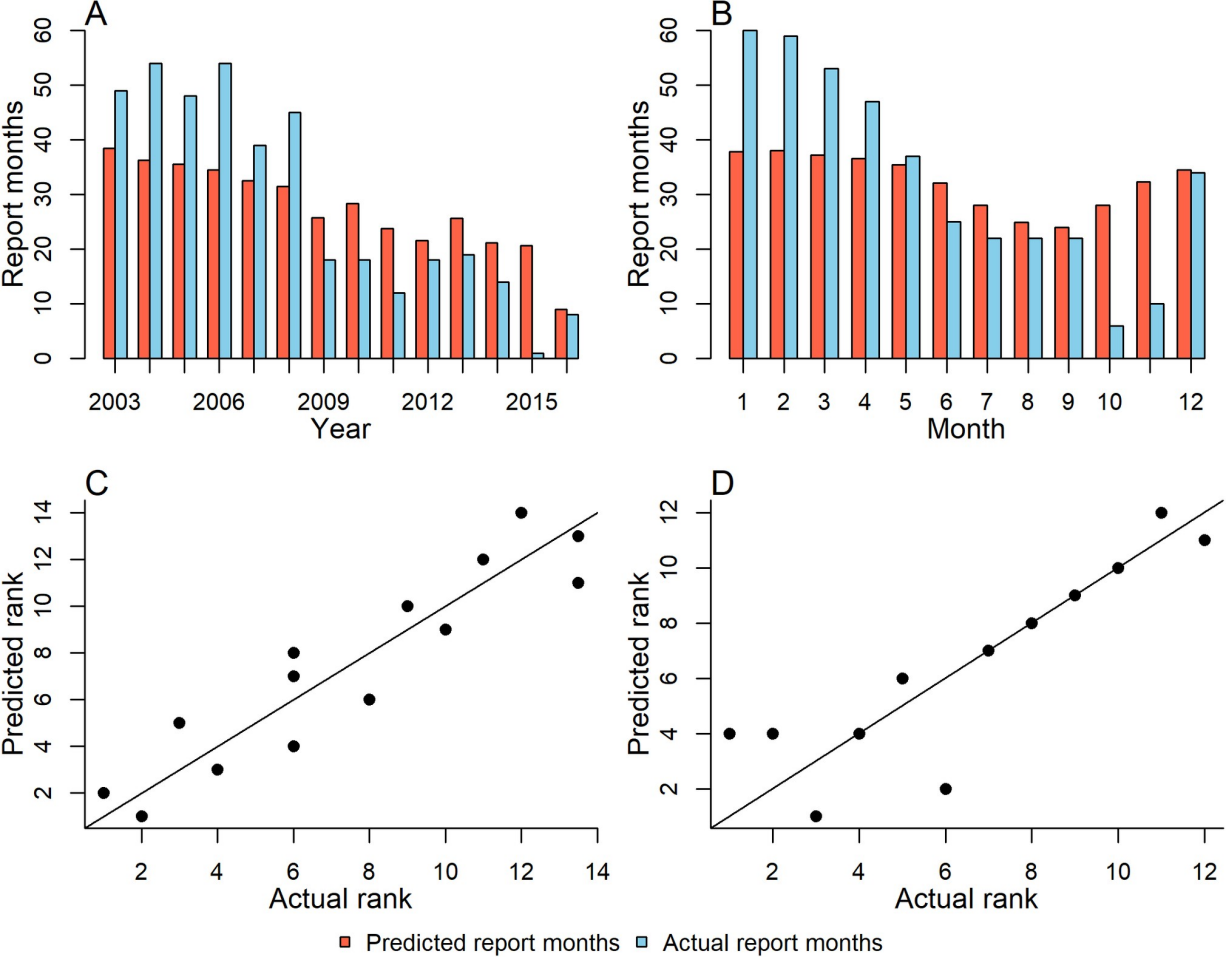
Summed ensemble model predictions (points) for (A) each year for the interannual model (A), and for each month for the seasonal model (B), contrast against the actual summed report months (lines) for each year or month. Yearly (C) and monthly (D) predictions ranked against the actual report months for the interannual and seasonal models, respectively (lines show predicted = actual).

In addition to representing geographic variation, the models also consider temporal heterogeneity in YF incidence (Fig 4). The interannual model and the seasonal model fit temporal trends with the in-sample R^2^ values of 0.43 (95% CI 0.41–0.45) and 0.66 (95% CI 0.64–0.67) respectively.

At the continent level, the inter-annual and seasonal model replicate the trends, but not the overall magnitude of temporal variation in report months. In the Inter-annual model, report months are underpredicted until 2009, after which they are slightly over-predicted. In the seasonal model, the model underestimates the data until the 5^th^ month, then over-predicts later months (Fig 4A and 4B). The accuracy of the models at the country level varies (see SI). When years and months are ranked by the number of report months, there is a high degree of concordance between predictions and the data. This is shown in the high Pearson correlation coefficient values between the predicted and actual rank of years and months, at 0.926 for the interannual model, and 0.873 for the seasonal model (Fig 4C and 4D).

### Drivers of seasonal, annual and long-term yellow fever transmission

The interannual and seasonal ensemble models showed both similarities and differences in the predictors found to be most significant (Table 2). For both, the covariate grouping relating to host demographics were the most important, with log of human population explaining the most variance in both model sets. The number of NHP species present also had a smaller but significant contribution for each. Both demographic predictors were positively associated with YF reports. The Enhanced Vegetation Index (EVI) was the second most important predictor for both models, again positively associated with YF reports. Other predictors differed between models, likely reflecting that the interannual model selected predictors best able to reflect long-term trends in YF reports, while the seasonal model selected those able to reproduce intra-annual seasonal patterns.

**Table 2:** Table of the permutation importance of different covariate groups, and individual covariates as well as standardised coefficient values. Only covariates that were significant in at least one of the model sets are shown. (A) Refers to the inter-annual model, and (B) the seasonal model.

Covariate groupings	% of models covariate group found in		Covariate	% of models covariate found in		Variable importance		Coefficient values	
	A	B		A	B	A	B	A	B
Climate	0	100	**Mean day temperature**	0	99.5	0	0.01		-2.71 (95% CI: -5.1 - -0.33)
			**Mean rainfall amplitude**	0	100	0	0.25		-0.35 (95% CI: -0.64 - -0.06)
Vegetation	100	100	**EVI**	100	100	0.82	0.84	6.42 (95% CI: 3.09–9.76)	6.21 (95% CI: 3.89–8.53)
			**EVI amplitude**	100	0	0.19	0	1.17 (95% CI: 0.33–2.01)	
Vegetation heterogeneity	100	0	**Vegetation heterogeneity**	100	0	0.79	0	3.34 (95% CI: 1.77–4.92)	
Vegetation heterogeneity temporal change	100	0	**Vegetation heterogeneity temporal change**	100	0	0.14	0	-0.39 (95% CI: -0.6 - -0.17)	
Land-cover	100	0	**Cropland cover**	100	0	0.21	0	-2.47 (95% CI: -4.27 - -0.66)	
			**Savanna cover**	100	0	0.5	0	-2.27 (95% CI: -3.73 - -0.8)	
Land-cover temporal change	100	0	**Savanna cover temporal change**	100	0	0	0	0.32 (95% CI: 0.1–0.54)	
Host demographics	100	100	**Number of NHP species**	100	100	0.48	0.74	1.34 (95% CI: 0.75–1.92)	1.68 (95% CI: 0.9–2.47)
			**Log of human population**	100	100	0.24	0.66	6.89 (95% CI: 0.62–13.16)	13.65 (95% CI: 5.19–22.11)

For the interannual model, landcover (cropland and savannah being negatively associated with YF) and vegetation heterogeneity (the standard deviation of EVI) were the next most important predictor groupings. Temporal changes between the current and previous year in vegetation and land-cover were significant predictors but made relatively small contributions to model fit. No climate coefficients were significant in the interannual model.

For the seasonal model, mean monthly day temperature and mean monthly rainfall amplitude (see Materials and Methods for definitions) were the other significant predictors, both negatively associated with YF reports.

Significant covariates were found in all (or almost) of the best performing models, with all covariate groupings found in interannual models except climate, and in the seasonal models only climate, vegetation and host demographics were found in the best performing models. Variable importance was highest in the EVI for both inter-annual and seasonal models, with the vegetation heterogeneity of a similar level of importance in the inter-annual model, and the number of NHP species and logarithm of human population slightly, but still important in the seasonal model. Despite the significance of the mean day temperature in the seasonal model, it was found to have an almost negligible variable importance–indicating it did not particularly contribute to predictive accuracy.

## Discussion

In this study we have described the geographic, seasonal and interannual trends in YF reports in Latin America from 2003–2016, using publicly available data. We used hierarchical negative binomial regression models to create ensemble models predicting interannual and seasonal variation in YF transmission with a series of climatic, land-cover, vegetation and host demographic covariates. Our models explained a substantial amount of the observed variation, with R^2^ values of 0.43 (95% CI 0.41–0.45) for the interannual and 0.66 (95% CI 0.64–0.67) for the seasonal model.

The geographic distribution of reports highlights “hotspots” for YF transmission, in Eastern Peru, North Western Peru and South Eastern Brazil (Fig 1B). The seasonal model reproduced these geographic trends more accurately than the interannual model. Continental-level interannual and seasonal trends in the data were also well-reproduced by the respective models, though both models captured geographic variation (e.g. at the country level) in these temporal trends less well (Figs 4 and S1 and S2)–albeit numbers of report months were often low when stratified by country. While at this level, the magnitude of temporal trends in report months are not fully captured, the relative ranking of years is and therefore model results can shed some light on what is associated with increased, or decreased, YF reporting in particular years and months.

While differing covariates are important for driving interannual and seasonal changes in YF transmission, vegetation (EVI) is highly influential for both models. This has been previously highlighted as a predictor of seasonal YF transmission [2], and potentially acts as a proxy for the interaction of rainfall and temperature, both important for arboviral transmission, while also taking into account a more complex interaction than is captured by either covariate alone. The potential additional complexity is highlighted through the absence of substantial correlations between either covariate and EVI. In both the interannual and the seasonal models, the log of human population was the most important predictor. This is not unexpected–larger populations give more opportunity for spillover, and since a report month is a month where one or more human YF cases are reported, larger populations are more likely to accumulate 1 or more cases in any one month even with a spatially invariant per-capita risk of YF. While there is no detected relationship between EVI and population at this spatial and temporal timescale, there is potentially an interaction of population and EVI, with anthropogenic pressures having long-term consequences for the EVI. However, at this spatial and temporal scale these changes in relation to YFV transmission are hard to disentangle.

For the interannual model, landcover and heterogeneity in vegetation were also influential covariates in explaining interannual variation in YF reports. While cropland and savanna cover are negatively associated with YF reports, vegetation heterogeneity is positively associated. The heterogeneity covariate we adopted maybe acting as a proxy for habitat fragmentation. Fragmentation may affect sylvatic hosts in a number of ways, such as increasing their exposure to human contacts via modified behaviours [50,51] or increased susceptibility to infection due to a stress-weakened immune system [52]. Furthermore, vegetation heterogeneity may alter vector dynamics and predispose greater rates of spillover either through increased human-sylvatic cycle contact or favouring of more anthropophilic vector species in fragmented habitats [53]. These effects have previously been suggested to affect zoonotic disease transmission, but until now had not been statistically implicated in YF emergence [14,31].

While we have explained a substantial proportion of the seasonal and inter-annual variation in YF reporting across South America (2003–2016) (Interannual model: 0.43 (95% CI 0.41–0.45), seasonal model: 0.66 (95% CI 0.64–0.67)), this still means that, respectively, 67% and 34% of this variation is unexplained. This, in part, may be due to the spatial resolution at which the study was carried out. Due to data limitations in the reporting of YF cases, we may not have fully captured the relationship between climate and environment with YF spill over at the local or individual level. This may explain why some covariates that may be expected to be associated with increased spillover, such as forest cover and change in forest cover, have not been found to be significant. Furthermore, these covariate changes may actually occur, and remain, over several years. By solely investigating year to year variation in the inter-annual model, we may not be accurately capturing the importance of these covariates by failing to find significant effects to what may be a significant relationship. Additionally, the usage of the calendar year, rather than a disease specific “transmission” based description of the year may lead to us to unable to find these associations of covariates with transmission. To account for this, future modelling work should take place at a higher spatial resolution and considering the role of multi-year variation in covariates, though the trade-off between the availability and quality of data with a potentially furthered understanding should be thoroughly explored.

While climatic and landcover fluctuations both inter- and intra-annually lead to changes that can lead to increased disease transmission, they do not represent the whole picture of spillover. In order for YF to enter human populations it has to be both circulating within the NHP reservoir, and there has to be human exposure to the sylvatic cycle. Across South America (2000–2014), 60% of human cases of YF were in people employed in farming, hunting or fishing–highly seasonal activities [36]. This changing risk of exposure is likely to account for a proportion of the temporal and spatial reporting of YF. In order to better capture these relationships with YF spillover into human populations across South America, future modelling exercises should endeavour to capture both the underlying suitability to disease transmission, and these correlates and determinants of exposure.

This analysis uses 397 months of YF report months, where we only included publicly available case reports [34,36] which had a confirmed onset date and which could be geolocated to at least the province level. Due to missing data, 23% of case reports were excluded from our analysis. In addition, due to the remote locations that sylvatic YF is often found in and the non-specific symptoms many cases show, it is likely that substantial numbers of YF cases are never recorded [15,54]. Underreporting in rural areas may lead us to underestimate YF risk in those locations. However, surveillance and data quality issues affect estimation of absolute case incidence, report months (presence/absence of cases in a specific administrative unit in a particular month) is likely to be more robust to under-ascertainment, as it only takes one reported case to be classified as YF positive. We are unable to identify whether the predictors of YF transmission we have identified affect sylvatic transmission or human exposure, given we have only analysed reports of human cases here. Data on NHP cases of YF across the continent are limited however, and their omission is a permissible oversight given this.

While expansion of the endemic zone is occurring, increases in population-level vaccination coverage in the endemic zones, where the majority of transmission is predicted, has precluded much of the human population from infection. This is in contrast with areas outside of this zone–where YF vaccination is either not usually necessary or not prioritised, and where spillover is more likely given the available of susceptible humans. This may go some way to explaining the decrease in report months over the time period (Fig 1).

In conclusion this body of work represents an important quantification of both the seasonality and interannual transmission of YF across South America (2003–2016). By identifying covariates, and their statistical relationship, with report months of YF, the work presented here may be used to highlight areas that have an increased probability for transmission. This may then allow for the targeting of surveillance in areas that have a higher risk of YF reporting, based on their climate and environment, without currently reported cases. This application could have substantial public health value, in a context where the geographic range of YF is changing and vaccine stocks are still limited.

## Supporting information

S1 Fig Inter-annual model predictions for the 8 countries reporting YF over the study period (2003–2016). The blue line indicates the data and the red dots the model predictions at the time-point.
(TIF)

S2 Fig Seasonal model predictions for the 8 countries reporting YF over the study period (2003–2016). The blue line indicates the data and the red dots the model predictions at the time-point.
(TIF)

S3 Fig A) The grid of 5° x 5° longitude of latitude with provinces assigned and colour coded by the grid point closest to their centroid coordinates. B) Examples of the training (blue) and validation (red) datasets as chosen by random sampling of grid points.
(TIF)

S1 Text Country-level inter-annual and seasonal model ensemble predictions. (DOCX)

S2 Text Out-of-sample validation: Spatial block bootstrapping. (DOCX)

S1 Data Dataset used for inter-annual models. (CSV)

S2 Data Dataset used for seasonal models. (CSV)
