# Supplementary figures and images for: Seasonal and inter-annual drivers of yellow fever transmission in South America
Source: PLoS Negl Trop Dis. 2021 Jan 11;15(1):e0008974. doi: 10.1371/journal.pntd.0008974 (PMC7822559; doi:10.1371/journal.pntd.0008974)

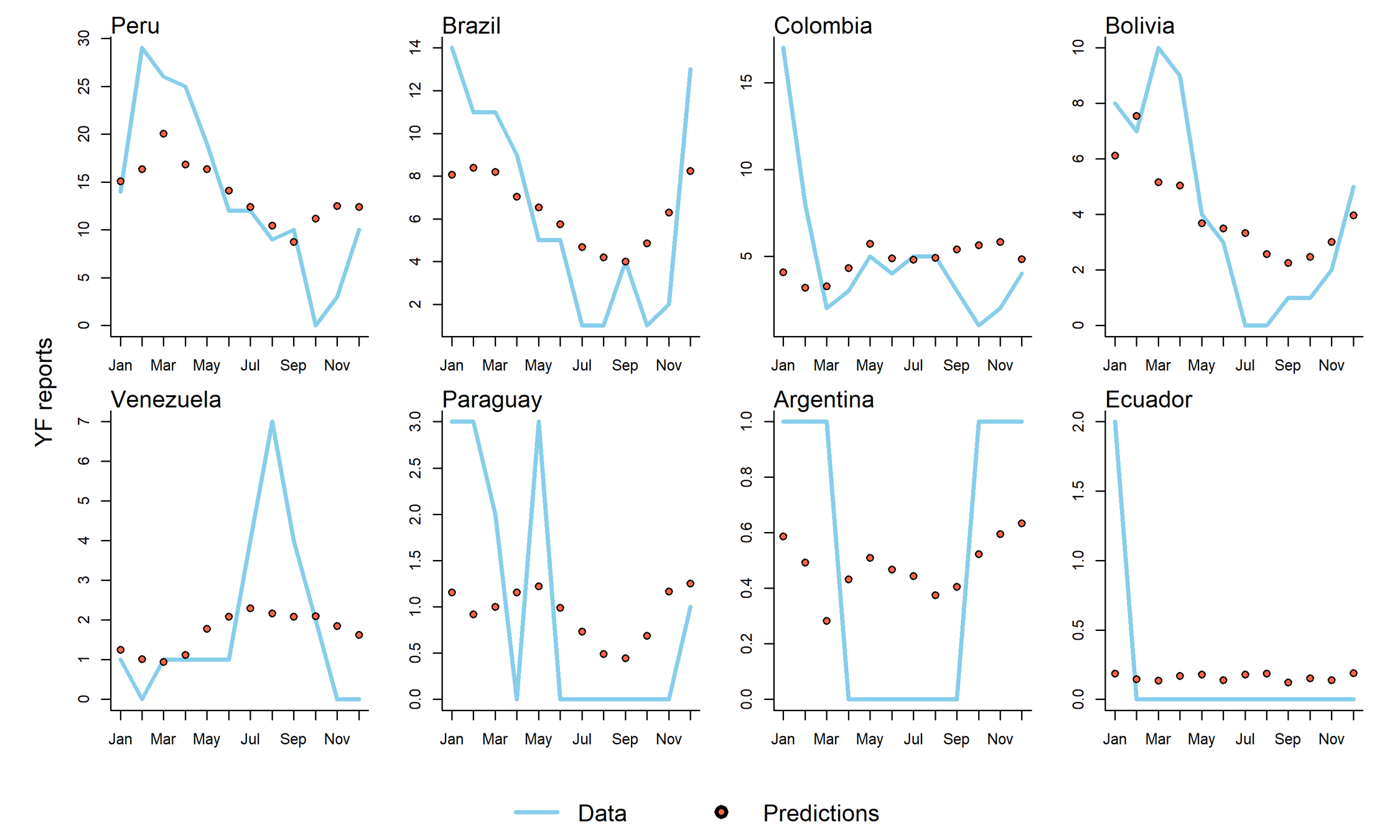

Supplement: S1 Fig — The blue line indicates the data and the red dots the model predictions at the time-point. (TIF) [file pntd.0008974.s001.tif]

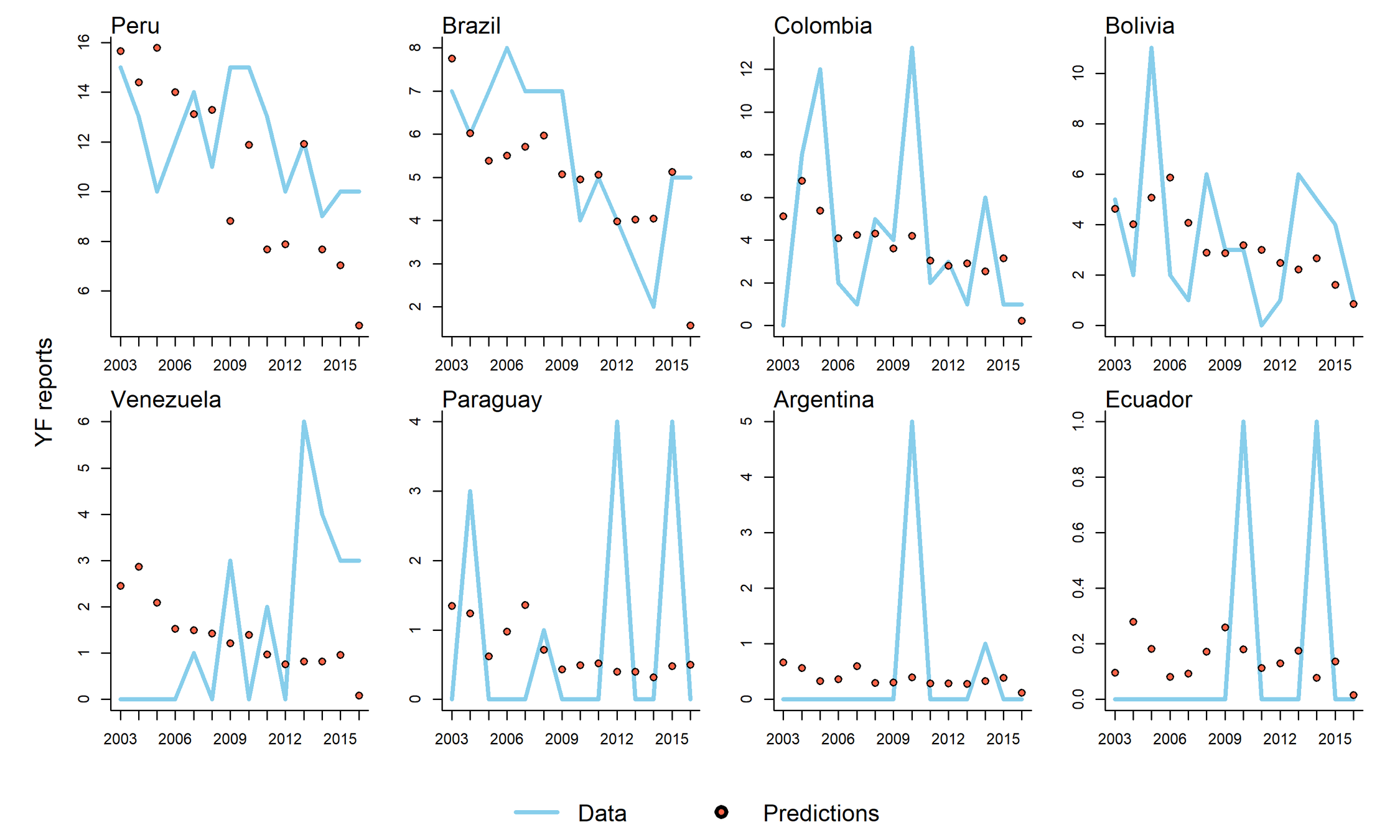

Supplement: S2 Fig — The blue line indicates the data and the red dots the model predictions at the time-point. (TIF) [file pntd.0008974.s002.tif]

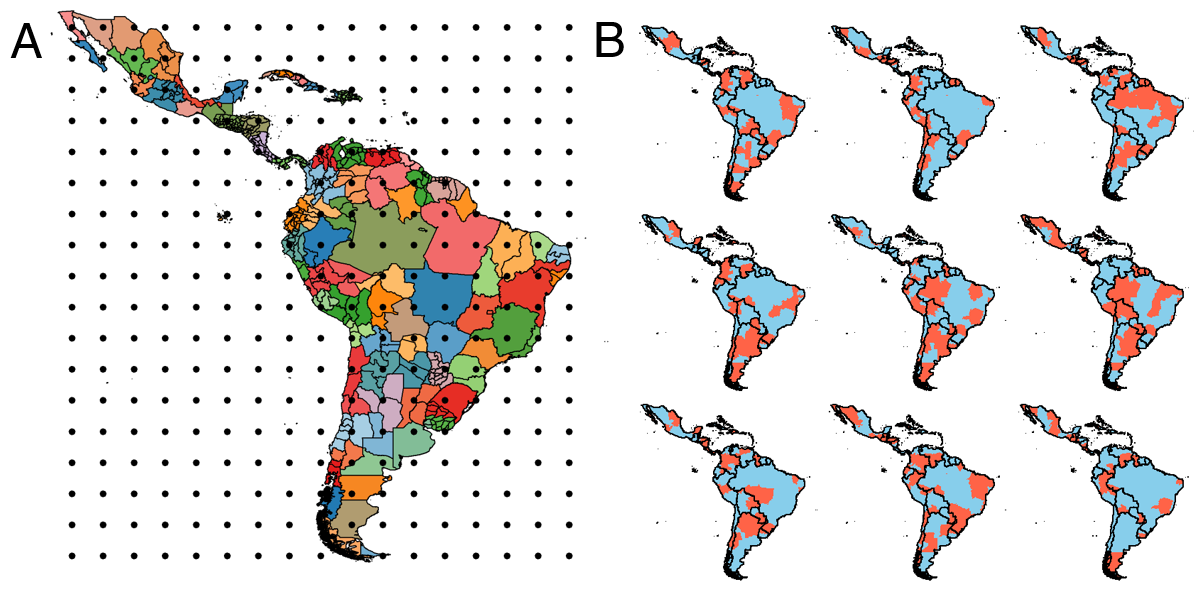

Supplement: S3 Fig — A) The grid of 5° x 5° longitude of latitude with provinces assigned and colour coded by the grid point closest to their centroid coordinates. B) Examples of the training (blue) and validation (red) datasets as chosen by random sampling of grid points. (TIF) [file pntd.0008974.s003.tif]
