# Supplementary material for: Seasonal and inter-annual drivers of yellow fever transmission in South America
Source: PLoS Negl Trop Dis. 2021 Jan 11;15(1):e0008974. doi: 10.1371/journal.pntd.0008974 (PMC7822559; doi:10.1371/journal.pntd.0008974)
Supplement: S1 Text — (DOCX) [file pntd.0008974.s004.docx]

**Country-level inter-annual and seasonal model ensemble predictions**These predictions are in-sample model predictions fit to all administrative units in question.

Inter-annual model predictions vary significantly between countries, with different fits relative to the underlying data (Figure 1). Generally the models over-predict the number of cases in all countries with confirmed cases, apart from Peru – where there is a significant underprediction.

While YF reporting often occurs in peaks separate by years of low transmission, our model predictions suggest that conditions are suitable for ongoing transmission, though at different levels of intensity, across the majority of countries in South America.


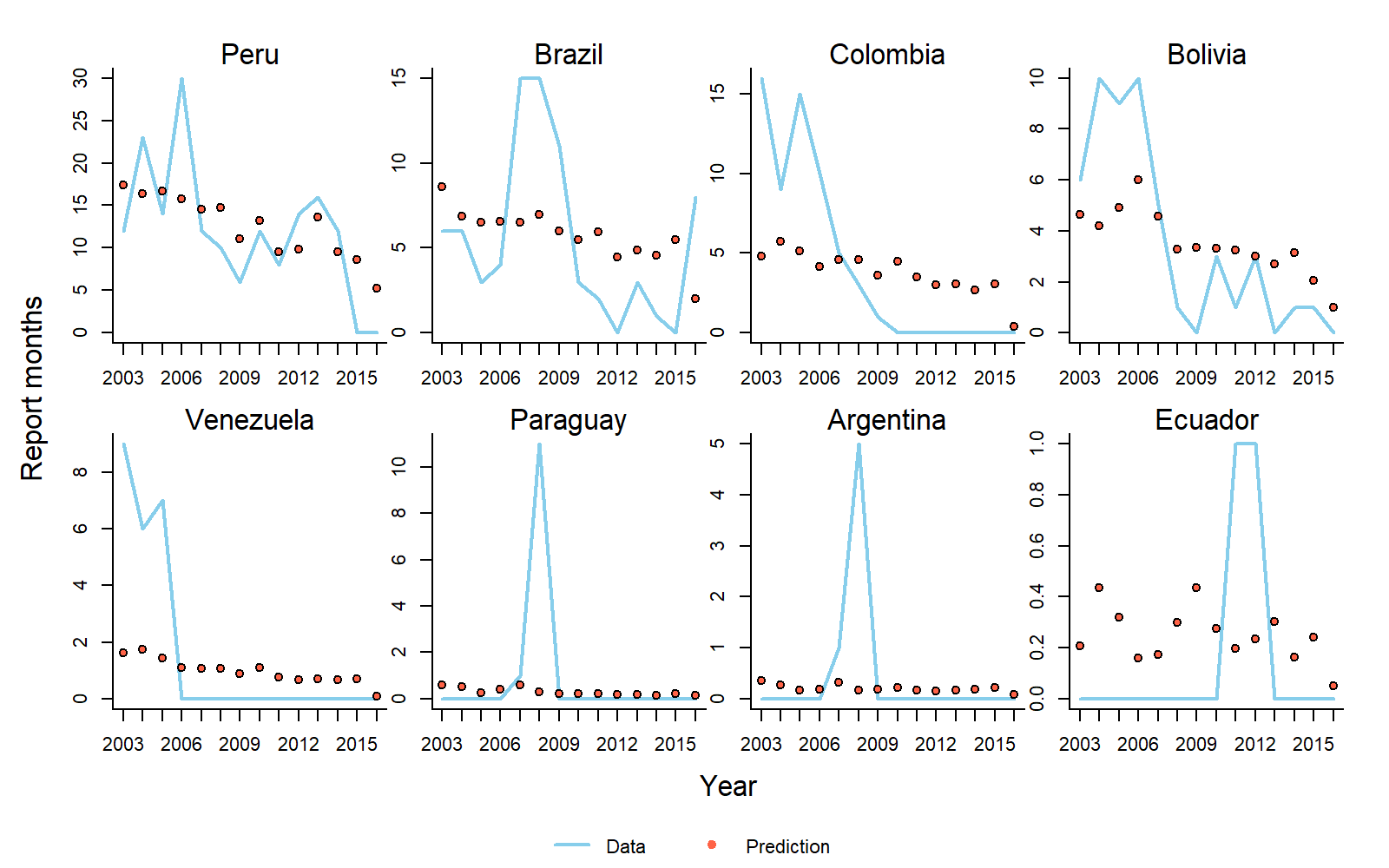


Figure 1. Inter-annual model predictions for the 8 countries reporting YF over the study period (2003-2016). The blue line indicates the data and the red dots the model predictions at the time-point.

YF seasonality varies substantially between countries, with the model capturing the trends, but not the overall magnitude of reporting. This leads to the model not capturing the true extent of seasonality – instead predicting some level of seasonality, but with reporting expected to continue throughout the year.

One explanation for this is that while there is a seasonality captured through our selection of covariates – potentially a greater level of seasonality in reporting is conferred through the seasonality of exposure. Occupational exposure through hunting, logging or farming may bring people into greater contact with the sylvatic cycle – something that is reflected in the majority of human cases in South America comprising of working age males [1].


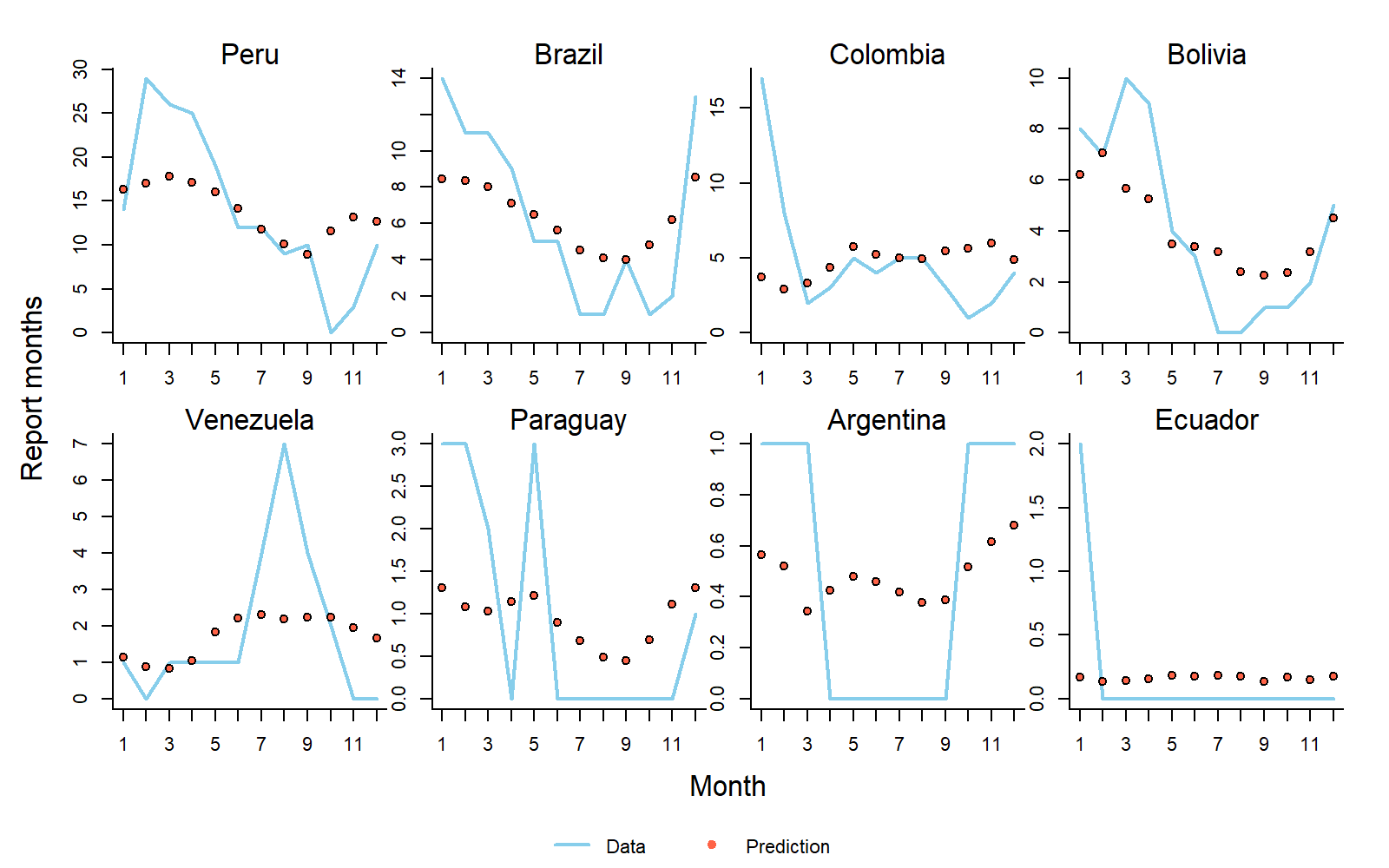


Figure 2. Seasonal model predictions for the 8 countries reporting YF over the study period (2003-2016). The blue line indicates the data and the red dots the model predictions at the time-point.

**References**

1. Pan American Health Organization. YELLOW FEVER: Number of Confirmed Cases and Deaths by Country in the Americas, 1960-2015 <http://ais.paho.org/phip/viz/ed_yellowfever.asp>: Pan American Health Organization,; 2017 [15/08/2017].
