# Supplementary material for: Seasonal and inter-annual drivers of yellow fever transmission in South America
Source: PLoS Negl Trop Dis. 2021 Jan 11;15(1):e0008974. doi: 10.1371/journal.pntd.0008974 (PMC7822559; doi:10.1371/journal.pntd.0008974)
Supplement: S2 Text — (DOCX) [file pntd.0008974.s005.docx]

**Out-of-sample validation: Spatial block bootstrapping**

To assess the out-of-sample predictive ability of our models we carried out a form of out-of-sample validation called spatial block bootstrapping.

This was done by overlaying a grid of 5° x 5° longitude of latitude over the study area and assigning provinces to a point based on their centroid coordinates (Figure 1). Following this, random sampling with replacement was used to build a training set of 60-70% of the points that contain an assigned province, the remaining unselected points were assigned to the validation dataset. This was repeated 200 times to generate 200 training sets and 200 validation sets.


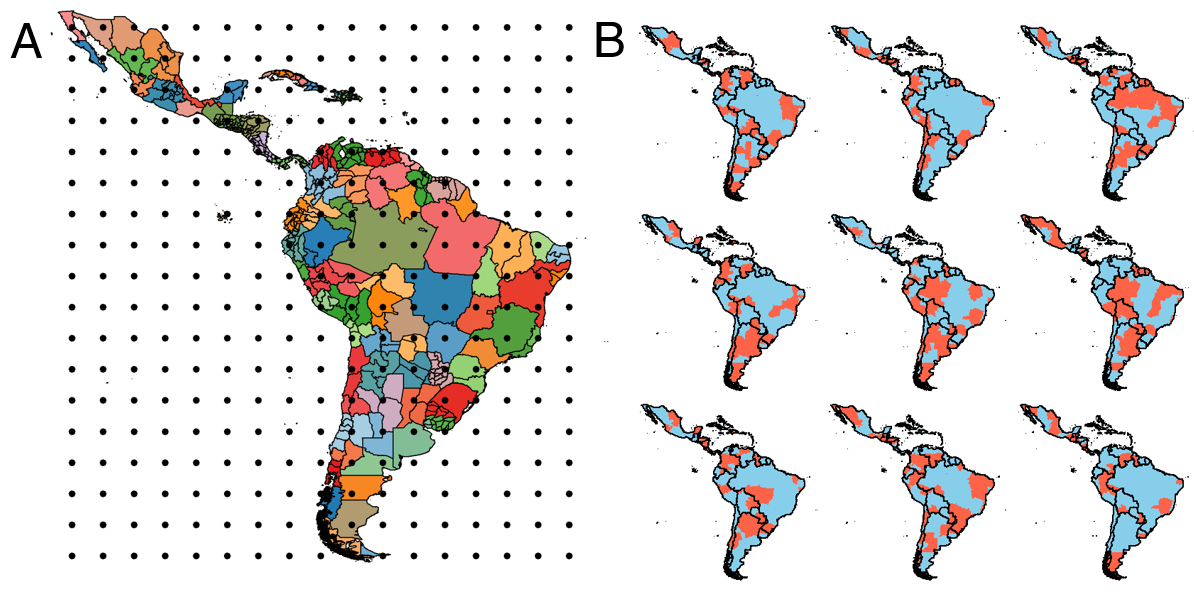


Figure 1. A) The grid of 5° x 5° longitude of latitude with provinces assigned and colour coded by the grid point closest to their centroid coordinates. B) Examples of the training (blue) and validation (red) datasets as chosen by random sampling of grid points.

The best performing models (as defined by models within 3 Akaike Information Criterion (AIC) of the lowest AIC) were then trained on the training set and predicted to the validation set. Out-of-sample performance was ascertained by the average coefficient of determination (R^2^). of model performance across all 200 runs.

These runs were then weighted and combined using their AIC value (see methods in main paper) and the out-of-sample performance calculated through their R^2^ value (Table 1). Given the robustness of this cross-validation method, the inter-annual and seasonal models performed well, particularly the inter-annual and seasonal models.

Table 1. Table showing the comparisons between in-sample and out-of-sample performance for the ensemble models.

| Model type | In-sample R^2^ | Out-sample R^2^ |
| --- | --- | --- |
| Inter-annual | 0.430 (95% CI 0.411 – 0.448) | 0.31 (95% CI 0.28 – 0.34) |
| Seasonal | 0.658 (95% CI 0.643 – 0.673) | 0.43 (95% CI 0.41 – 0.45) |
